# How to Make a Do-It-Yourself, Disposable Bite Guard Using Easily Available Materials, to Prevent Tongue and Lip Injuries, During Motor Evoked Potential Monitoring in Neurosurgery

**DOI:** 10.7759/cureus.5536

**Published:** 2019-08-30

**Authors:** Gopalakrishnan M Sasidharan, Bujji Karre

**Affiliations:** 1 Neurosurgery, Jawaharlal Institute of Postgraduate Medical Education and Research (JIPMER), Pondicherry, IND

**Keywords:** motor evoked potentials tcemep, bite injury, bite block, do-it-yourself, iatrogenic injury, intraoperative neurophysiological monitoring, tongue injury, bite guard, lip injury, tongue bite

## Abstract

We describe a do-it-yourself method of making a bite guard, using pairs of Foley catheters and surgical gloves to prevent tongue, lip, and other injuries during the monitoring of transcranially elicited motor evoked potential. We have used it in five cases, and have found that the hack is particularly cost-effective and reliable. We describe the technique here using multiple photographs.

## Introduction

Intraoperative neurophysiological monitoring is increasingly used as a standard adjunct to prevent inadvertent neurological injury during cranial and spinal surgical procedures. Tongue and lip injuries due to bites while eliciting transcranial electrical stimulation for recording intraoperative motor evoked potential (TcMEP), have an incidence ranging from 0.2 to 0.63 % [1]. Rarely, even fractures of the incisor teeth have been described [1]. Correct placement of an appropriately sized bite guard protects against this hazard to a great extent, though there is no consensus on the number or configuration of bite guards that need to be placed. Though disposable and reusable bite guards are available commercially, they may not be easily available in many parts of the world. In many centers, including our center, neurophysiologists and anesthesiologists have tried to use other materials like rolled gauze pieces, or syringes wrapped in gauze as bite guards, and have encountered suboptimal and unreliable performance [2]. Injuries can occur if these materials are kept between the incisor teeth as they can get displaced, or because the sides of the tongue remain unprotected [3]. Though frequent intraoperative checking has been suggested, this is impractical when the patient has been draped for cranial neurosurgery or if the patient is in a prone position. An injured tongue can swell up in the postoperative period and cause pain and discomfort while eating food. In the worst-case scenario, a swollen tongue can obstruct the airway.

## Technical report

**Figure 1:**
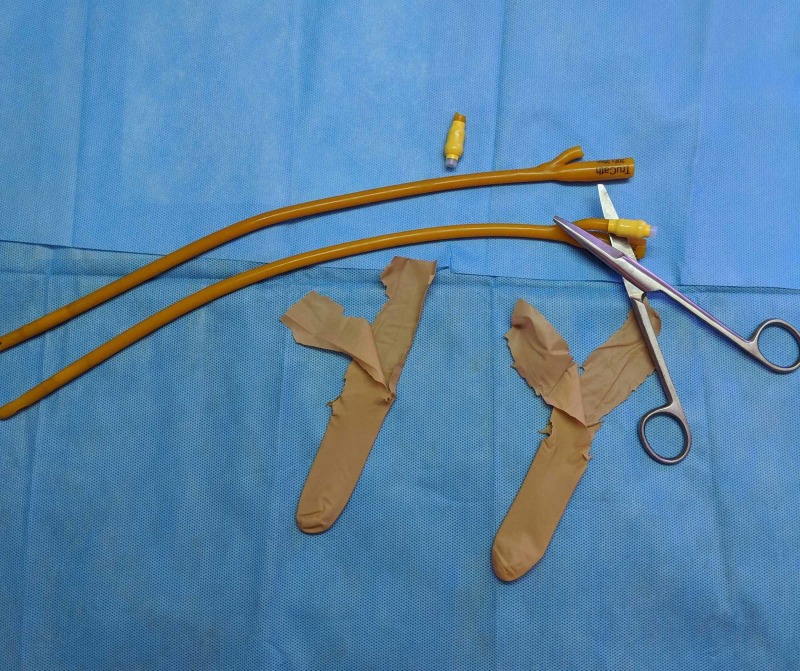
The materials required are two Foley catheters and the cut fingers of a surgical glove.

**Figure 2:**
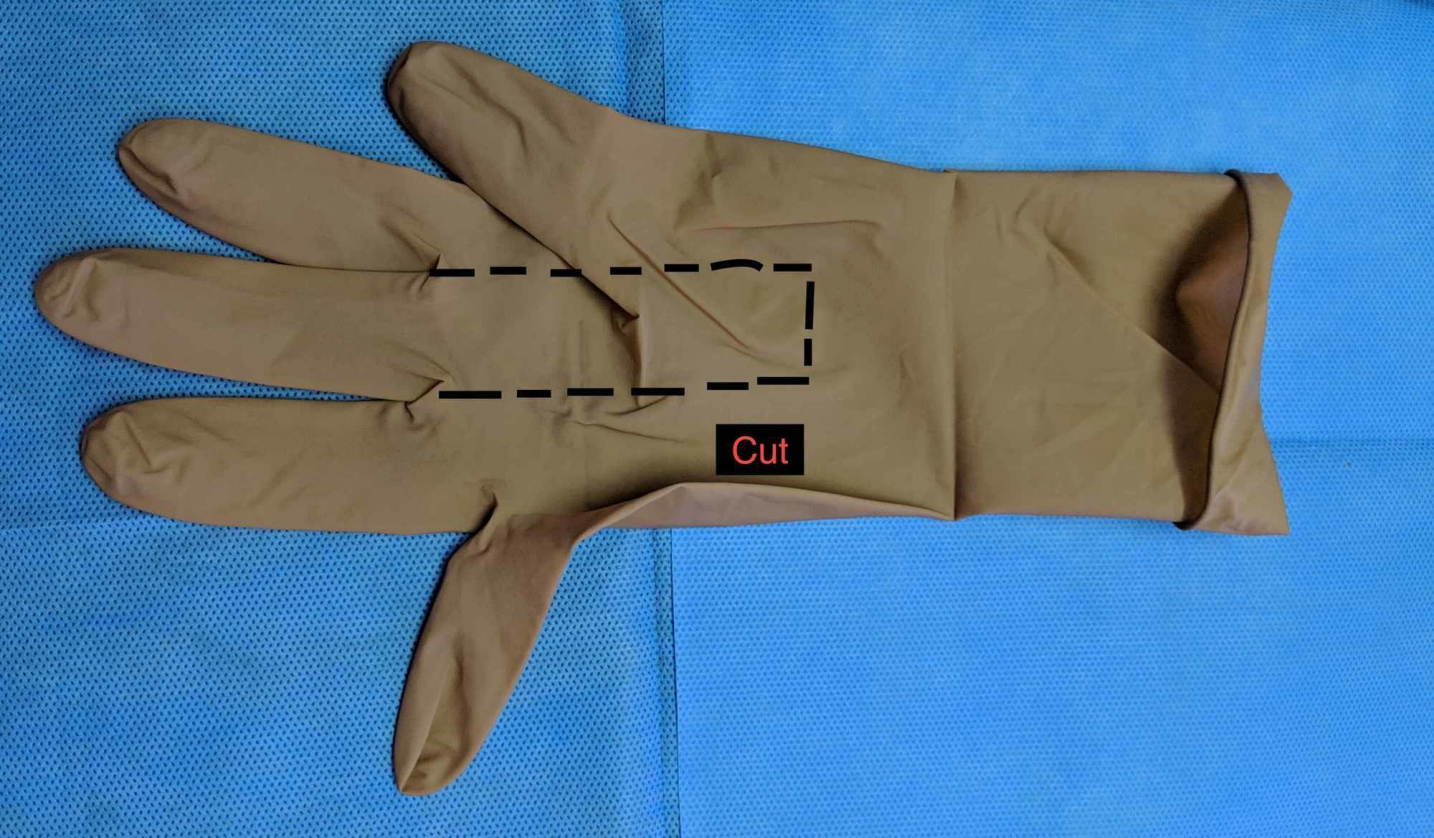
A finger of the surgical glove is cut as indicated by the dotted line, to leave two loose flaps.

Herein, we describe an easy method of making disposable bite guards using commonly available materials, to prevent tongue, lip, and teeth injuries. The only materials required are two 16F (or 18F) Foley catheters, and a pair of surgical gloves, as shown in Figure 1. The first step involves cutting out two fingers of the surgical gloves along the dotted line, so that there are two free and loose flaps for each, as illustrated in Figure 2. While cutting the flaps, the edges may become ragged, but it is not of any consequence.

**Figure 3:**
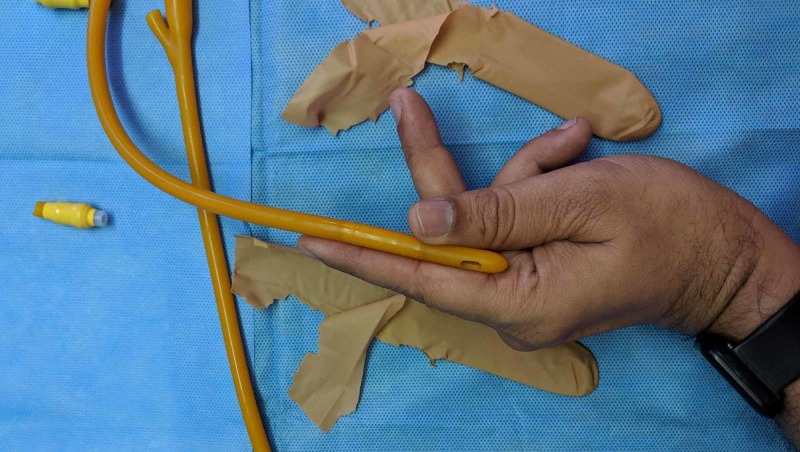
The index finger can be used as a rough measure to loop the catheter for about 7 cm.

**Figure 4:**
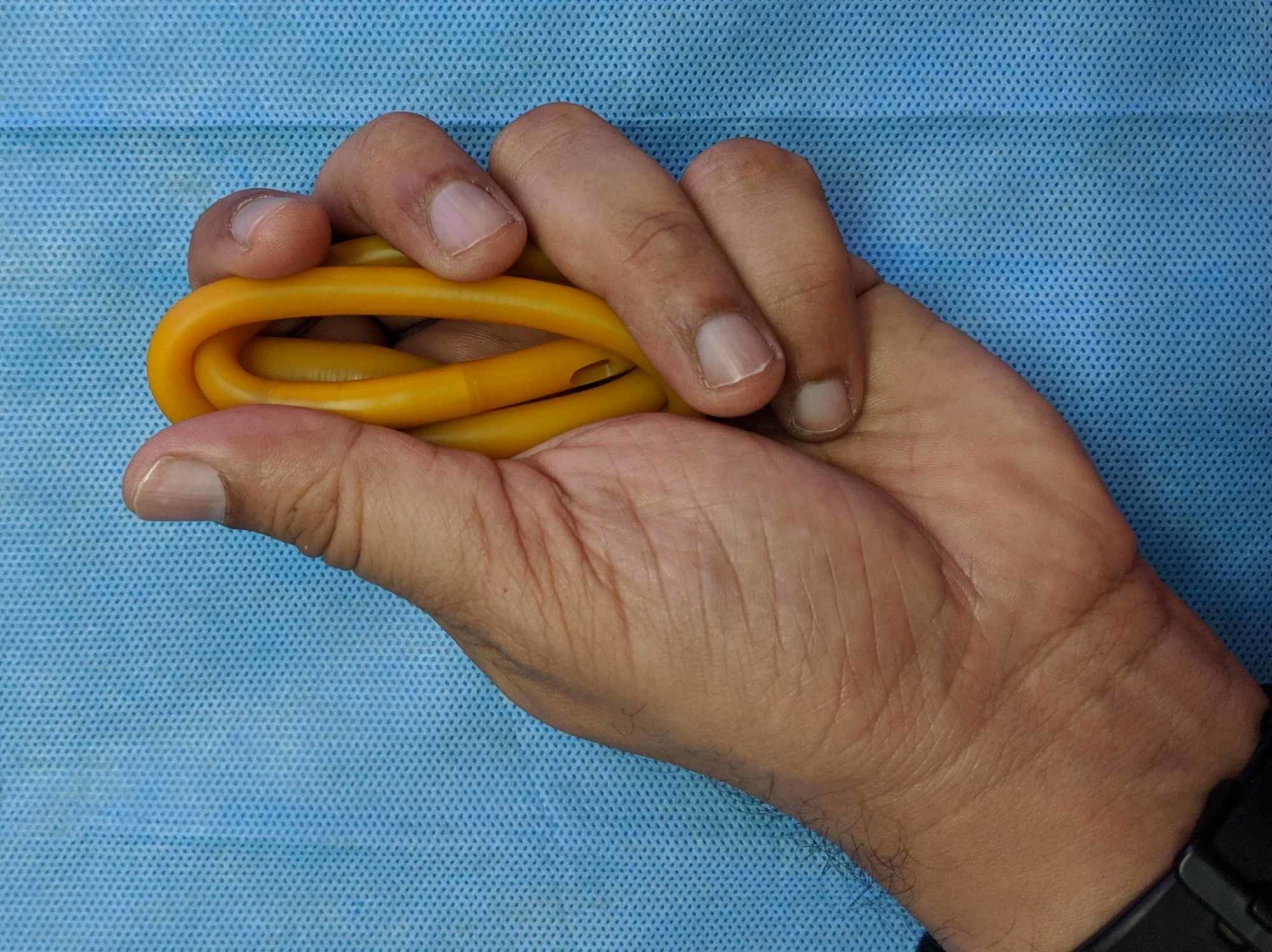
Looping the catheter over itself, in preparation for inserting it into the cut glove finger.

**Figure 5:**
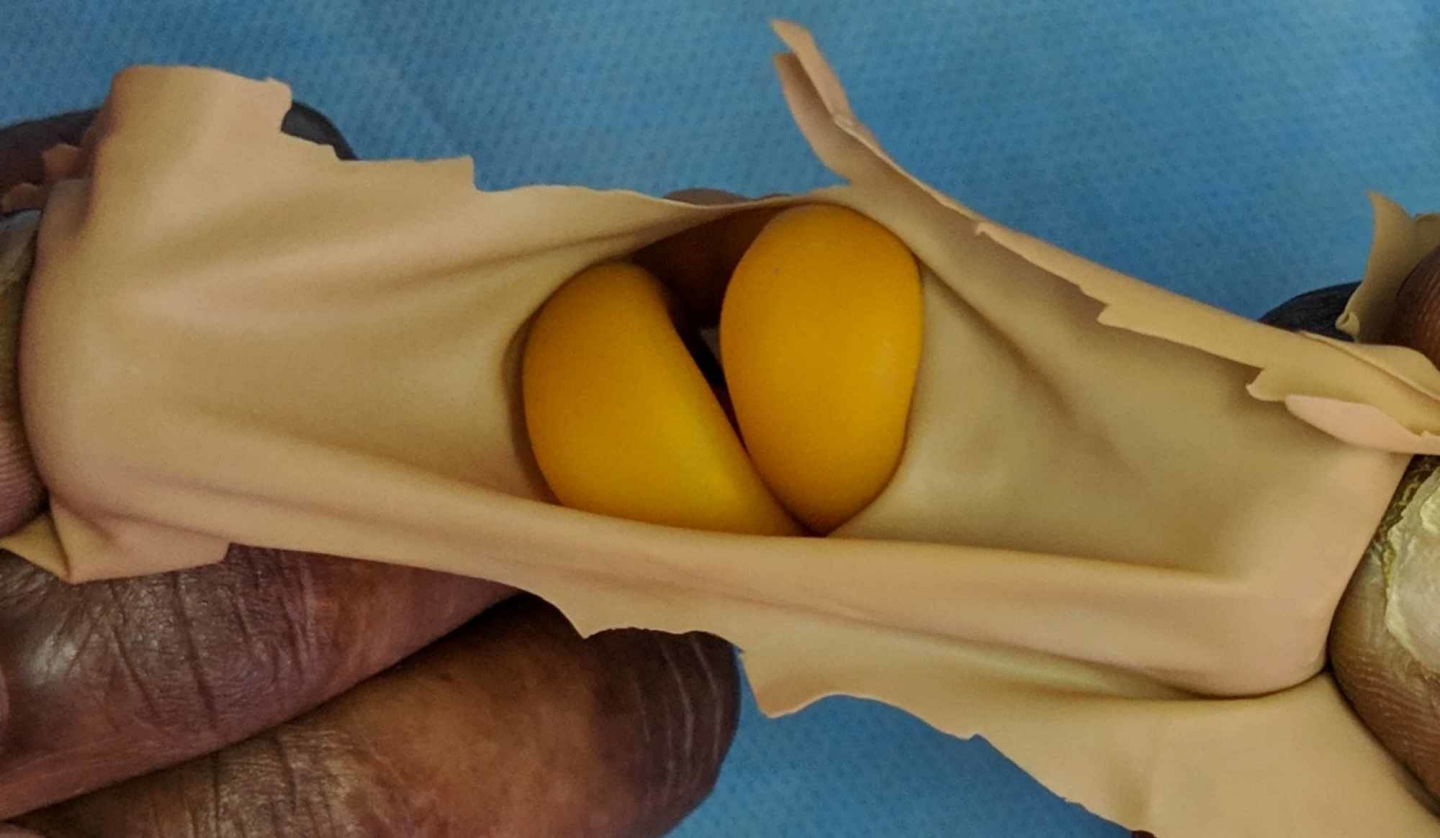
The looped catheter is inserted as shown, while the mouth of the cut finger of the glove is held open by another person.

**Figure 6:**
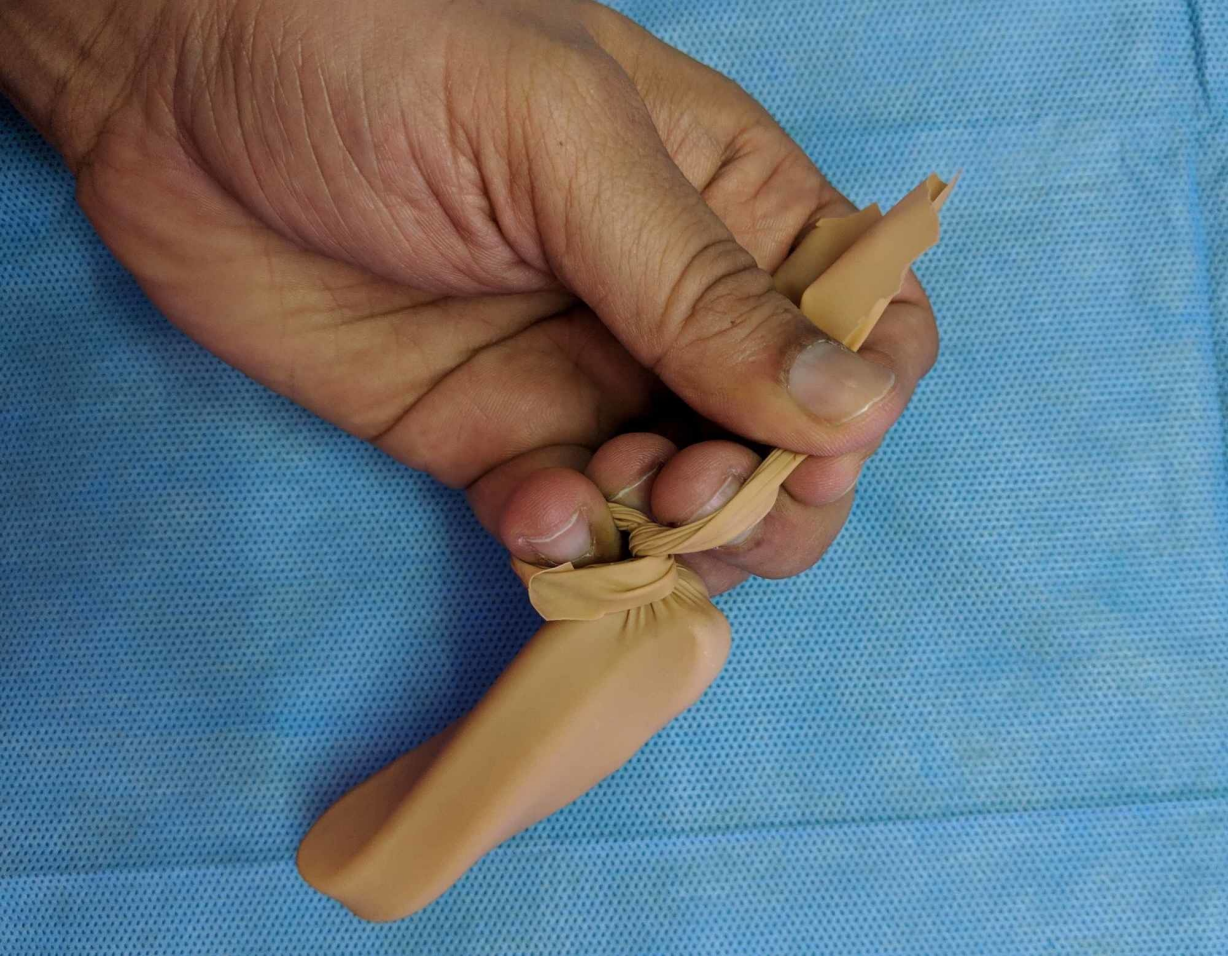
The free ends of the glove finger are tied together to keep the loop inside

**Figure 7:**
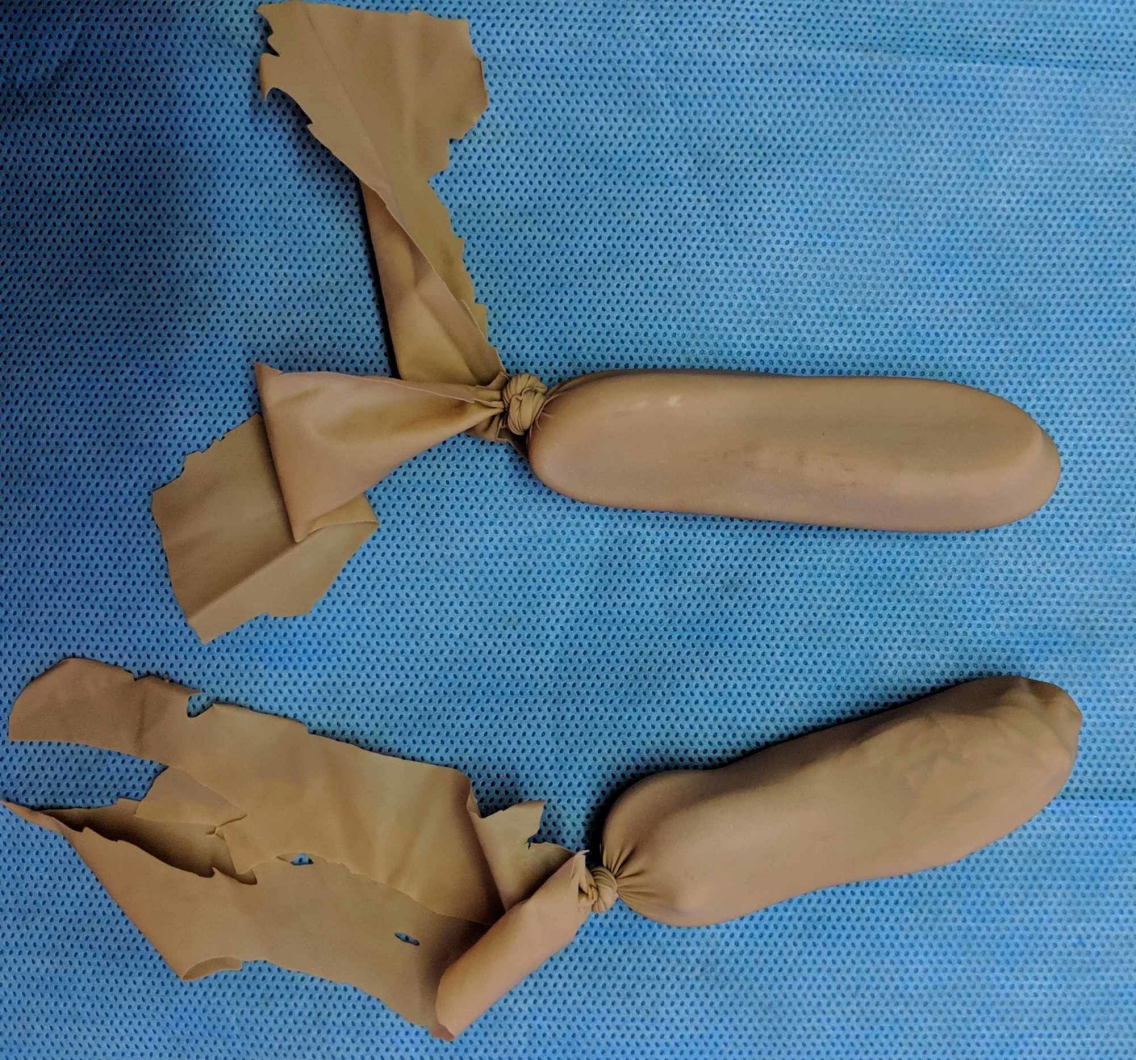
The finished products should look like these.

The next step is to cut off the hard valve of the catheter and loop the catheter to a length of about 7cm. The index finger can be used as a rough measure, as shown in Figure 3. The loop is then inserted into the cut ‘finger’ of the glove, and the proximal parts of the free ends are tied together. The free ends are left loose to get the final product, as shown in Figures 4-7.

**Figure 8:**
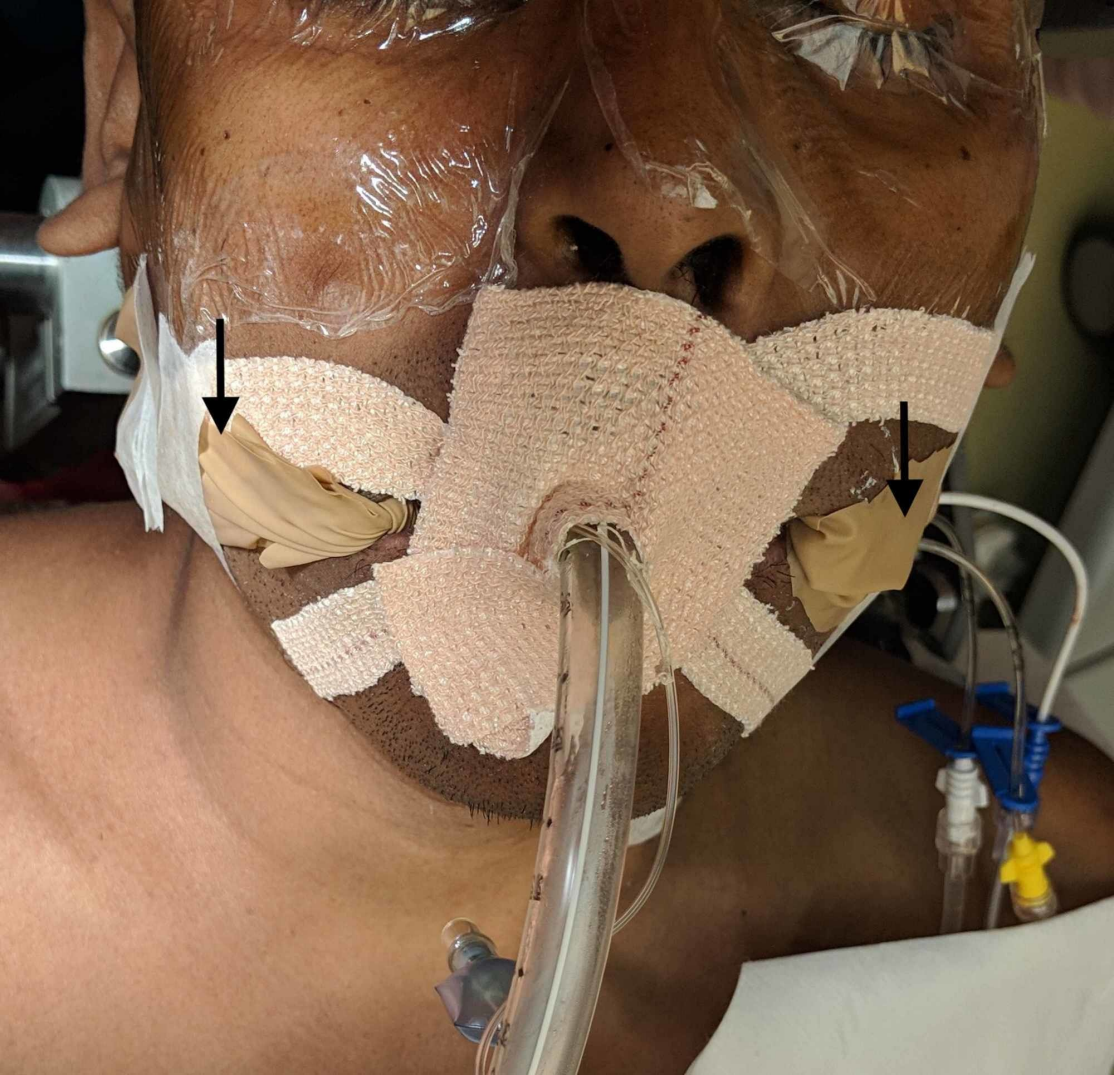
The loose, free ends are taped to the cheek as shown by the arrows.

The two bite guards are inserted between the molar and premolar teeth on both sides. The space within the loops and the elastic nature of the wound Foley catheter tightly contained within the glove finger allow a snug fit between the molar teeth and absorb the bite force admirably. It also displaces the tongue away from the teeth edges. The two free ends are taped to the outside of the cheeks for safe retrieval at the end of surgery. The endotracheal tube is secured at the center of the mouth, as shown in Figure 8.

## Discussion

We have utilized this method of protecting the tongue and lips, in five patients in whom TcMEP monitoring was used. Typically it takes about five minutes or less to make two bite-guards and requires no special technical skill. While the baseline TcMEP is acquired before draping the patient, the correct placement of the bite guard can be verified by checking the approximating movement of the jaws, and ensuring that the incisor teeth remain separated during the clenching motion. We have encountered no injuries so far, though the number of cases we have used it in, is few. The bite guard was not damaged due to the bite-force in any patient, indicating the resilience of the construct.

**Figure 9:**
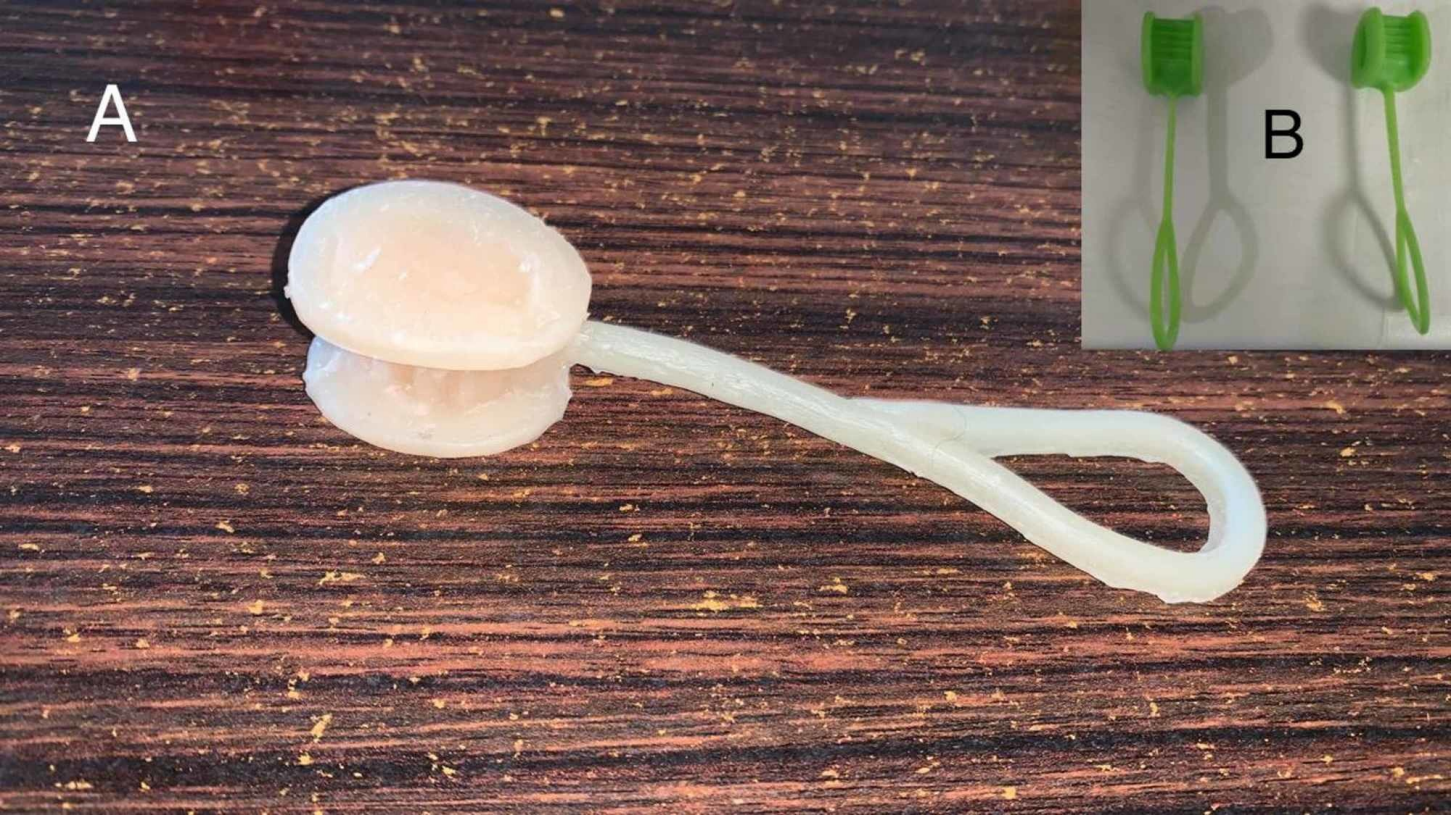
A hand-molded silicone bite block (A), and a commercially available pair in the inset (B)

A recent report by Yata et al. suggests that the incidence of bite-induced iatrogenic injuries may be much higher than previously reported, and can be as high as 6.5% when carefully looked for by oral surgeons [4]. TcMEP causes direct and forceful contraction of the muscles of mastication, especially when C3 and C4 electrodes according to the 10-20 system are used for stimulation. Although we did make bite guards similar to those commercially available, using medical-grade silicone, as shown in Figure 9, the performance of the bite guard described here, was found to be as efficient.

This bite guard made from a glove and a Foley catheter can be made easily by a health care worker in any part of the world, unlike silicone molded bite guards which require specialized workmanship. Though there are no prospective trials reported on the configuration of bite guards which provide the best protection, our experience indicates that a pair of sufficiently long and bulky bite blocks as described here, kept between the upper and lower rows of molar-premolar teeth, works well. An additional guard may be placed between the incisors for extra protection, though we did not use a third guard in any patient.

There are certain advantages to this technique, in that the cost is minimal, and the bite guard can be disposed of, instead of the off-label sterilization often practiced when using imported or costly, commercially available bite blocks. It is also possible to make customized bite guards by changing the size of the Foley, the length, or the number of the loops, to correctly fit between the upper and lower molar teeth, according to a particular patient’s anatomy.

We suggest the use of latex-free gloves and 100% silicone Foley catheter if latex allergy is an issue.

## Conclusions

The do-it-yourself hack described here can be used to quickly and easily make a bite guard, when an off-the-shelf device is unavailable. It effectively prevents bite injuries while monitoring transcranial motor evoked potentials in cranial and spinal surgeries, though the device needs to be tested in larger numbers of patients to ensure its reliability.

## References

[1] Tamkus A, Rice K (2012). The incidence of bite injuries associated with transcranial motor-evoked potential monitoring. Anesth Analg.

[2] Hao TJ, Liu G, Ang P (2014). A rare complication of tongue laceration following posterior spinal surgery using spinal cord monitoring: a case report. Indian J Anaesth.

[3] Williams A, Singh G (2014). Tongue bite injury after use of transcranial electric stimulation motor-evoked potential monitoring. J Anaesthesiol Clin Pharmacol.

[4] Yata S, Ida M, Shimotsuji H (2018). Bite injuries caused by transcranial electrical stimulation motor-evoked potentials’ monitoring: incidence, associated factors, and clinical course. J Anesth.

